# Is Age a Limiting Factor for Audiological Results in Active Middle Ear Implants?

**DOI:** 10.3390/jpm13121650

**Published:** 2023-11-26

**Authors:** J. Manuel Morales-Puebla, Luis Lassaletta, Isabel Sánchez-Cuadrado, Miryam Calvino, Javier Gavilán

**Affiliations:** 1 Department of Otolaryngology, La Paz University Hospital, IdiPAZ Research Institute, 28046 Madrid, Spain; llassaletta@salud.madrid.org (L.L.); isabelpilar.sanchez@salud.madrid.org (I.S.-C.); miryam.calvino@salud.madrid.org (M.C.); javier.gavilan@salud.madrid.org (J.G.); 2 Biomedical Research Networking Centre on Rare Diseases (CIBERER), Institute of Health Carlos III, U761, 28029 Madrid, Spain

**Keywords:** active middle ear implant, vibrant soundbridge, hearing loss, elderly, quality of life

## Abstract

Active middle ear implants (AMEI) are implantable options for patients with sensorineural, conductive, or mixed hearing loss who are not good candidates for hearing aids. The aim of this study was to compare audiological, surgical, quality of life, and sound quality outcomes in adults <60 and ≥60 years receiving an AMEI. Twenty adult patients who underwent AMEI implantation were divided into two groups, <60 and ≥60 y. Preoperative tests included pure-tone average and speech discrimination score (SDS) at 65 dB for disyllabic words in quiet. Postoperative measures included AMEI-aided bone conduction threshold, free-field warble-tone threshold, and SDS at 65 dB for disyllabic words in quiet 12 months after the AMEI fitting. Subjective benefit was evaluated using the Nijmegen Cochlear Implant Questionnaire (NCIQ), Glasgow Benefit Inventory (GBI), and Hearing Implant Sound Quality Index (HISQUI_19_). Mean functional gain was 32 and 30 dB, and SDS at 65 dB improved from 19 to 95% and from 31 to 84% in the <60 and ≥60 y groups, respectively. All NCIQ domains improved following surgery, and all patients had a positive overall GBI score. The mean HISQUI_19_ score was 97 in both age groups. AMEIs are an effective hearing restoration method for older adults suffering from conductive or mixed hearing loss.

## 1. Introduction

The treatment of conductive and mixed hearing loss has changed since the appearance of active middle ear implants (AMEI). These devices transform sound energy into mechanical energy which is transferred to the ossicles of the middle ear or in the case of conductive and mixed hearing loss directly to the oval window (OW) or round window (RW). They are an alternative to traditional reconstructive middle ear surgery, bone conduction implants, or hearing aids when these options are not suitable for the patient or do not offer sufficient audiological benefit [1,2].

**Figure 1:**
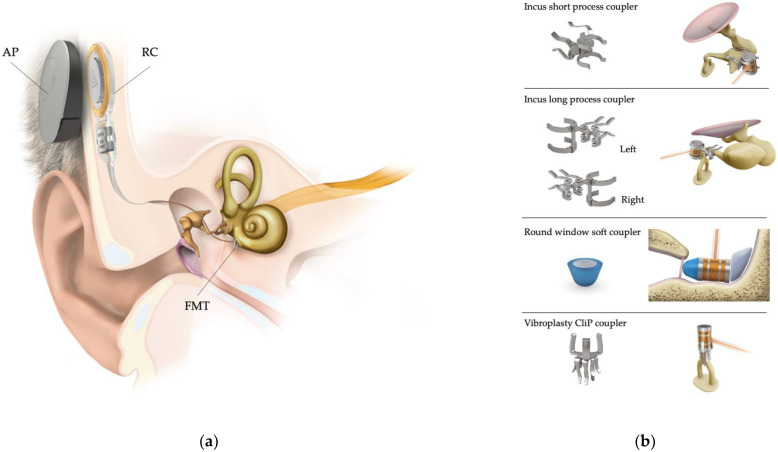
Vibrant Soundbridge. (**a**) Semi-implantable device. AP: audio processor, RC: receiver coil, FMT: Floating Mass Transducer. (**b**) Most commonly used coupling options for the FMT. There are also available: vibroplasty-bell-coupler, vibroplasty-OW-coupler, and vibroplasty-RW-coupler, not shown in this figure. (Courtesy of Med-El Co., Innsbruck, Austria; with permission.).

At the present time, the only AMEI available is the Vibrant Soundbridge (VSB). It is a semi-implantable device with an external audio processor and an implantable component called the Vibrating Ossicular Replacement Prosthesis (VORP). The VORP consists of two parts: the receiver coil and the Floating Mass Transducer (FMT) [3]. The audio processor and the receiver coil are coupled magnetically, with intact skin between them. The acoustic signal is received by the external audio processor which converts it to an electric signal that is transferred via radiofrequency to the receiver coil. In the middle ear, the FMT can be positioned attached to the ossicular chain or directly on the RW [2] (Figure 1). Restoring hearing loss by means of middle ear vibratory stimulation with an AMEI is named vibroplasty [2].

**Figure 2:**
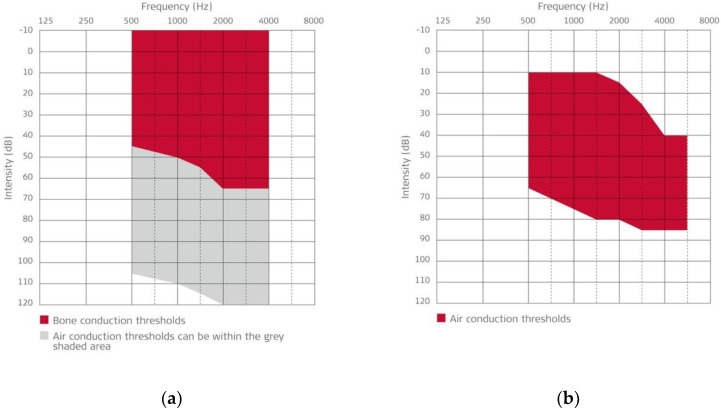
(**a**) Vibrant Soundbridge indication for conductive or mixed hearing loss: bone conduction thresholds must fall within the shaded area in the chart. (**b**) Vibrant Soundbridge indication for sensorineural hearing loss: air conduction thresholds must fall within the shaded area in the chart. In both cases, speech understanding of at least 50% at the most comfortable level with headphones in open-set word test is required. (Courtesy of Med-El Co., Innsbruck, Austria; with permission.).

Although initially designed for sensorineural hearing loss with the FMT coupled to the long process of the incus [3], the VSB has also been demonstrated to be an effective alternative for conductive and mixed hearing loss when the FMT is coupled to the stapes or on the windows of the middle ear [4,5,6]. Audiological indications for the VSB are shown in Figure 2. It can be implanted in patients with normal anatomy or who have had previous middle ear surgery, with or without an available ossicular chain. It represents a versatile implantable device to consider, in addition to conventional hearing aids and bone conduction implants, for the personalized treatment of hearing loss.

According to the United Nation’s World Population Prospects (2019 revision), by 2050, 25% of the population in Europe and North America could be aged 65 or over [7]. It is estimated that about 25% of people from 65 to 75 years old and 70% to 80% of people over 75 years old suffer from age-related hearing loss [8]. In this scenario, an AMEI is the main implantable option for senior patients with sensorineural or mixed hearing losses who are not good candidates for hearing aids. Cochlear implantation in elderly people has been questioned, and no differences have been found in auditory gains and quality of life (QOL) compared with younger adults [9]. It has not been established yet if there exists an age limitation for VSB.

This study aimed to compare the audiological, surgical, QOL, and sound quality outcomes in adults younger and older than 60 years of age who received an AMEI.

## 2. Materials and Methods

### 2.1. Study Design

A retrospective study of consecutive AMEI candidates from February 2008 to May 2022 was conducted in the Department of Otorhinolaryngology of a tertiary hospital. The study procedures were approved by the Ethics Committees (PI-1544).

Participants were enrolled in the study if they met the following inclusion criteria: conductive or mixed hearing loss with VSB implantation and over 18-years of age.

Written, informed consent was obtained from all subjects. The procedure involved an audiological evaluation and a series of questionnaires about the perceived benefit of AMEI and QOL. Audiological evaluations were conducted twice: just before implantation and 12 months after the first AMEI fitting. QOL questionaries were evaluated 12 months after the AMEI fitting. All participants were implanted with a VSB (MED-EL GmbH, Innsbruck, Austria). Continued implant usage at the end of the study was registered.

Patients were divided into two age groups: <60 years (<60 y) and ≥60 years (≥60 y). This cut-off was previously established in other studies that evaluated age-related hearing loss [10,11].

### 2.2. Procedures

#### 2.2.1. Audiological Assessment

Audiological assessment was performed in a double-walled, soundproof booth using a two-channel Madsen Astera2 audiometer (Otometrics, Taastrup, Denmark). Preoperative tests included pure-tone air and bone conduction thresholds at 500, 1000, 2000, and 4000 Hz and the maximum Speech Discrimination Score (SDS) for disyllabic words in quiet under unaided conditions. Postoperatively, measures included pure-tone bone conduction thresholds at 500, 1000, 2000, and 4000 Hz, sound field warble-tone thresholds at 500, 1000, 2000, and 4000 Hz, and the SDS for disyllabic words in quiet with the VSB on. Participants were seated 1 m away from the loudspeakers at 0° azimuth. The functional gain was defined as the mean difference between unaided preoperative pure-tone air conduction thresholds and aided warble tone thresholds [12]. Postoperative air bone gap (pABG) was measured as the difference between aided warble tone thresholds and unaided postoperative pure-tone bone conduction thresholds. The speech discrimination tests were performed without lip reading, at 65 dB SPL, with phonetically balanced disyllabic words from the test developed by Cárdenas de and Marrero [13]. The results were presented as percentages.

#### 2.2.2. Surgical Procedure

VSB was implanted in 20 patients. Eight <60 y patients and seven ≥60 y patients underwent vibroplasty with placement of the FMT in the RW, as described by Colletti et al. [14]. In the younger group, the FMT was located on the stapes in two cases and on the short process of the incus in another one. In the older group, there was one case on the stapes and one case on the short process of the incus. Cases implanted on the short process of the incus had an intact ossicular chain, the first one with blunting after myringoplasty and the other one with external auditory canal stenosis (Table 1). The surgical technique for VSB on the RW has been described elsewhere, both for standard and radical mastoidectomy and subtotal petrosectomy [15,16,17].

#### 2.2.3. Questionnaires

Subjective benefit with VSB was evaluated using the Spanish version of the Nijmegen Cochlear Implant Questionnaire (NCIQ), Glasgow Benefit Inventory (GBI), and Hearing Implant Sound Quality Index (HISQUI_19_) tests.

The NCIQ questionnaire was completed twice: before implantation and at least one year after the first VSB fitting. The GBI and HISQUI_19_ questionnaires were completed only after implantation.

The NCIQ is a validated, closed-set questionnaire [18] comprising 60 items. It was developed to evaluate the health effects of cochlear implant use. It has three general domains: physical, psychological, and social functioning. Each domain is divided into subdomains. The physical domain consists of basic sound perception, advanced sound perception, and speech production; the social domain consists of activity and social functioning; and the psychological functioning domain has only one subdomain—self-esteem. Each item includes a statement with a five-point response scale to indicate the degree to which the person finds the statement to be true. Although initially designed for cochlear implants, this questionnaire has been applied to AMEIs in other studies [12,19].

The GBI is a validated questionnaire developed to retrospectively assess the QOL after otorhinolaryngologic interventions [20]. It consists of 18 questions and generates a scale from −100 (maximum detriment) through 0 (no change) to +100 (maximum benefit). It assesses an individual’s perception of the overall success of VSB use in terms of social and physical functioning (“Overall Benefit”, “General Health”, “Social Support”, and “Physical Health”).

The HISQUI_19_ is a validated questionnaire [21] used to determine sound quality in everyday listening situations. It consists of 19 items with a seven-point Likert scale (1—“never” to 7—“always”). The scores of individual items are added together to produce a total score. A total score of 19–29 indicates very poor sound quality, 30–59 poor sound quality, 60–89 moderate sound quality, 90–109 good sound quality, and 110–133 very good sound quality.

#### 2.2.4. Data Analysis

To compare audiometric data and self-reported outcomes (NICQ, GBI, HISQUI_19_) between the age groups (<60 y and ≥60 y) at two time points (before and after implantation), the *t*-test (when the data were normally distributed) or the Mann–Whitney U test were used. To measure the difference within the groups, the *t*-test (when the data were normally distributed) or the Wilcoxon test were used. Normality was assessed with the Shapiro–Wilk test and Q-Q plots.

Pearson’s correlation coefficient was independently calculated for the <60 y and the ≥60 y groups to evaluate the relationship between age, audiometric data (PTA4 and speech perception test results), and questionnaire scores (NICQ, GBI, HISQUI_19_).

Missing data and the response option “Not applicable” were treated as missing values. A level of *p* ≤ 0.05 (two-tailed) was considered significant. Statistical analyses were performed in the SPSS software package v24.0 (IBM Corp., Armonk, NY, USA).

Demographic characteristics and outcome measures are presented as absolute values, percentages, and, where appropriate, the mean and ± SD are provided.

## 3. Results

### 3.1. Participants

Twenty adult participants with conductive or mixed hearing loss undergoing VSB implantation were enrolled in this study: <60 y (n = 11, mean age = 45 ± 10 years) and ≥60 y (n = 9, mean age = 67 ± 9 years). The demographic data of both groups are presented in Table 1.

### 3.2. Surgical Outcomes

All patients underwent surgery uneventfully. Chronic ear with previous middle ear reconstruction was the most frequent indication. In the older group, two patients had unusual indications, including hearing rehabilitation following an infratemporal approach for a paraganglioma, and a subtotal petrosectomy to seal cerebrospinal fluid (CSF) leak. Table 2 shows the FMT coupling locations in all patients.

### 3.3. Audiological Assessment

The unaided PTA4_AC_ and SDS scores before implantation in both age groups are shown in Table 1. After implantation, all participants used their audio processors daily. Postoperative audiometric tests outcomes are presented in Table 2.

**Figure 3:**
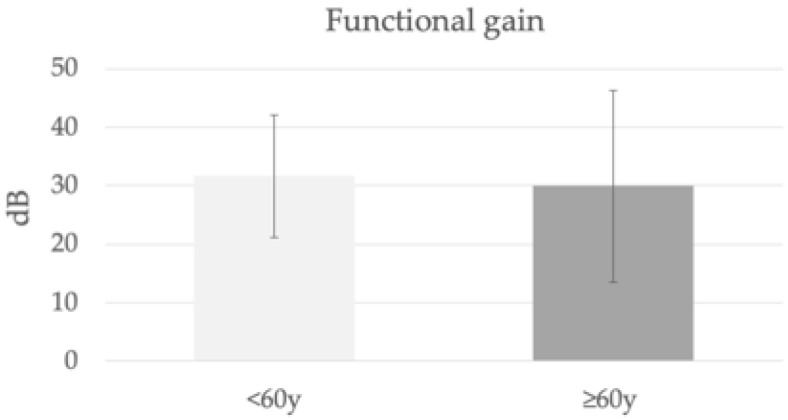
Functional gain one year after Vibrant Soundbridge activation in both age groups.

**Figure 4:**
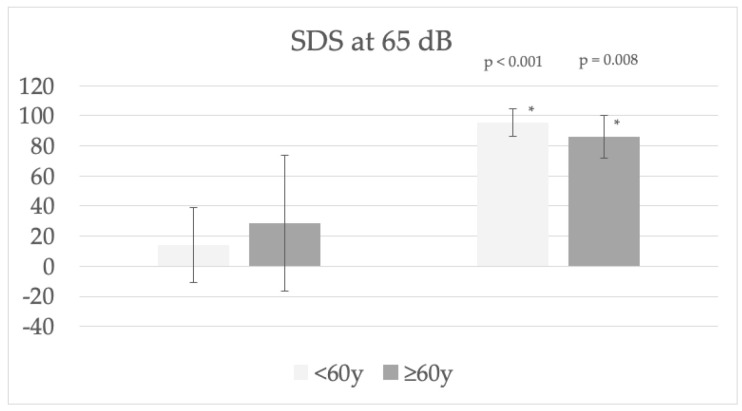
Speech discrimination score (SDS) before and one year after Vibrant Soundbridge activation in both age groups. * Statistically significant, *p* < 0.05.

The mean functional gain one year after activation of the audio processor was 32 and 30 dB (Figure 3), (PTA4 improved from 66 to 34 dB (*p* < 0.001) and from 73 to 43 (*p* = 0.001)), SDS at 65 dB improved from 19 to 95% (*p* < 0.001) and from 31 to 86% (*p* = 0.008) in the <60 y and ≥60 y groups, respectively (Figure 4). Mean functional gain was also compared between RW and ossicular chain coupling, with no differences between the two options.

The mean follow-up was 8 years (1–14). All participants used their audio processor daily during follow-up and by the end of the study.

### 3.4. Subjective Questionnaries

**Figure 5:**
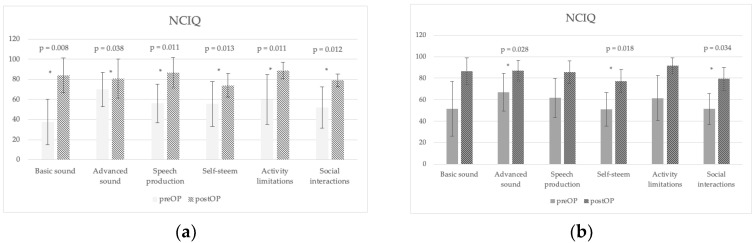
Preoperative (preOP) and postoperative (postOP) Nijmegen Cochlear Implant Questionnaire (NCIQ) results in the <60 y group (**a**) and in the ≥60 y group (**b**). * Statistically significant, *p* < 0.05.

All NCIQ domains improved following surgery after 12 months of VSB use, both in younger and older implantees. The greatest benefits were observed in basic sound perception and in speech production scores in the <60 y group, from 38 to 86 and from 56 to 89, respectively. In the ≥60 y group, the greatest benefits were observed in advanced sound perception, self-esteem, and social interactions, from 67 to 89, from 51 to 73, and from 51 to 76, respectively. There were no significant differences between age groups (Figure 5).

The overall GBI score was positive in both age groups (mean of 61 and 33 in <60 and ≥60 y, respectively) (Table 3). When combining both groups, 100% of participants reported a positive overall change after VSB implantation. There were no significant differences in the overall GBI scores and the subscale scores in both groups.

**Figure 6:**
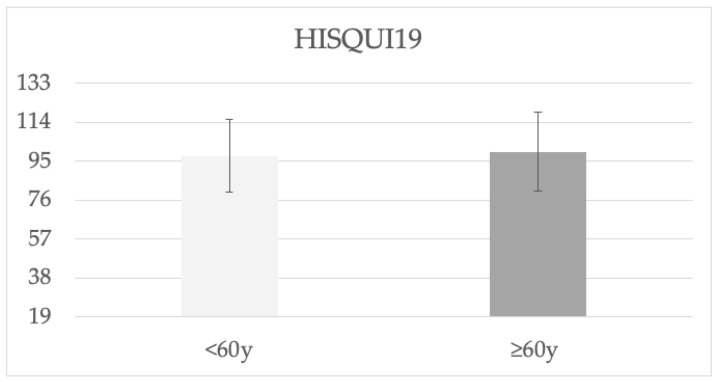
Hearing Implant Sound Quality Index (HISQUI_19_) after Vibrant Soundbridge implantation in in both age groups. Total score achieved: <30 = very poor sound quality, 31–60 = poor sound quality, 61–90 moderate sound quality, 91–110 = good sound quality, 111–133 = very good sound quality.

After VSB implantation, both age groups obtained “good” sound quality, with 97 and 100 mean HISQUI_19_ scores in the <60 y and ≥60 y groups, respectively (Figure 6).

### 3.5. Relationship between Age and Audilogical and Subjective Benefit Outcomes

No correlation was found between age, audiological outcomes, and the QOL questionnaires.

## 4. Discussion

This study comparing age-related outcomes with the VSB confirms that this AMEI is an effective solution for mixed or conductive hearing loss regardless of patient age. After VSB implantation, all patients improved their audiometric thresholds, with a mean functional gain of 32 and 30 dB and a pABG of 1 and −6 dB. SDS at 65 dB improved from 19 to 95%, and from 31 to 84% in the <60 and ≥60 y groups, respectively. All implanted patients continue using their audio processors daily.

Improvements were also registered in speech perception, perceived sound quality, and QOL using the NCIQ, GBI, and HISQUI_19_ questionnaires.

### 4.1. Audiological Outcome with the VSB

The first objective of this study was to evaluate audiological results after VSB implantation. The PTA4 and SDS score improved significantly in both age groups. Our outcomes are similar to other published series [2,22,23,24].

The direct transmission of the vibration generated by the implant to the ossicular chain or the cochlear fluids provides good functional gain, especially at high frequencies. This improvement in functional gain is associated with less distortion and feedback than hearing aids, therefore leading to better speech understanding [1,25]. Tysome et al. [26] published similar results in a systematic review comparing AMEIs with conventional hearing aids. In their review, it was highlighted that AMEIs offered better functional gain and improvement in speech perception in noise as well as higher patient satisfaction due to better sound quality, less feedback, and no external auditory canal occlusion [26].

It has been proposed that FMT RW stimulation could be the result of bone conduction instead of direct transmission to the inner ear, but it has been demonstrated that the FMT is too small and not powerful enough to generate vibrations of sufficient strength to produce a bone-conducted stimulation [27].

Some authors have suggested that positioning the FMT on the RW is associated with worse coupling efficiency and therefore less favorable audiological outcomes than other FMT positioning options [28]. Although ossicular coupling provides the best results [23,29], it has been demonstrated that if the FMT is directly attached to the RW good functional outcome can also be achieved [30]. FMT RW coupling quality can be optimized by intraoperative electrocochleography registry; by doing so, the surgeon can decide whether to place the FMT directly on the RW membrane or interpose tissue between the FMT and RW, depending on the intraoperative response [12]. Currently, electrocochleography has been replaced by examining auditory brainstem responses [31]. By using this method, the FMT position can be modified and fixed based on where the best wave V identification is achieved. In our patients, better functional gain results were obtained with FMT RW coupling in the older group, whereas no differences were found in the younger group when compared with ossicular chain coupling.

Good audiological results with the VSB, stable over time, have also been published irrespective of the FMT coupling position [2,6,32,33]. Functional gain in our study (32 dB in the <60 y group and 30 dB in the ≥60 y group) was lower than the 43 dB reported by Zahnert et al. [34] or the 36 dB reported by Rhane et al. [2] with the FMT positioned on the RW. Nevertheless, the SDS score in our patients, 95% and 86% (<60 y and ≥60 y groups, respectively), was higher than that of Rahne et al. at 73% and Zahnert et al. at 73.3%, although this can be due to the use of bisyllabic words in our speech discrimination test [2,34]. All patients included in this study are daily users of the implant with a mean follow-up of 8 years.

Several studies have demonstrated that patients’ postoperative bone conduction thresholds remains unchanged compared with the preoperative hearing situation, which suggests that inner ear auditory function is not affected by RW vibroplasty [24,25,33]. In our study, the <60 y group showed a postoperative increase in bone conduction thresholds due to bone conduction changes of more than 10 dB observed in one stapes FMT coupling and three RW surgeries. The ≥60 y group showed a postoperative increase of bone conduction thresholds in two RW surgeries. These patients, in both groups, still benefited from the VSB when comparing pre- and postoperative PTA and SDS at 65 dB. Rahne et al. [2] also reported on VSB benefits despite changes in bone conduction thresholds, and, although they did not distinguish between age groups, their two patients were over 60 years old. These two cases were implanted with a short process incus coupler, and the authors determined that the bone conduction changes were unrelated to the surgery or the device. Some authors have stated that hearing loss after middle ear surgery could be related to ossicular chain manipulation, acoustic trauma due to the bone drilling [35], perilymphatic fistulae after manipulation of the oval or round window, or perioperative middle ear infections associated with labyrintitis [36].

### 4.2. VSB and Surgical Outcomes

The second objective was to evaluate surgical outcomes. The VSB surgery and immediate postoperative period were successful in both patient groups in our study (younger and older than 60 years of age). Despite the fact that older individual tend to have more comorbidities and risks associated with anesthesia [37], in our patients, age had no influence on the surgical outcome.

The ossicular coupling of the FMT is less surgically challenging [29], but the ossicular chain is usually interrupted or missing in patients with chronic otitis media. In the absence of an ossicular chain, oval window vibroplasty can be an alternative [22], although RW vibroplasty is performed most often [6,12,16,17,24,25].

RW vibroplasty commonly requires RW niche drilling because of the high anatomical variability of the area. This poses an added risk of iatrogenic severe sensorineural hearing loss [29] due to accidentally opening the RW membrane. The careful identification and exposure of the RW membrane must be performed [17]. Most of our patients had RW FMT coupling, with no profound hearing loss observed and successful implant usage in all cases.

Two patients in the ≥60 y group had special indications (CSF leak and paraganglioma). Surgery in these cases was also uneventful despite the greater surgical complexity.

Patients were offered an active transcutaneous bone conduction implant as an alternative to the VSB if their bone conduction thresholds were in its range of indication. However, if bone conduction thresholds in high frequencies are over 55 dB, the only transcutaneous implantable device for conductive or mixed hearing loss would be the VSB. Considering that bone conduction thresholds are usually worse in the elderly population, the VSB could be their best implantable option. Not only have we not found differences between the <60 y and ≥60 y groups, but RW vibroplasty also remains a good alternative for social hearing restoration in the older age group [38,39].

### 4.3. Effect of the VSB on Subjetive Outcomes

The third objective of our study was to assess QOL and sound quality outcomes. Considerable changes in self-reported QOL questionnaires were reflected by the postoperative improvement of all NCIQ domains, the positive GBI scores, and the good sound quality perceived on the HISQUI_19_ by all patients.

No significant differences were found for the NCIQ and HISQUI_19_ in both age groups.

Edlinger et al. [32] established a positive correlation between SDS benefit at 65 dB and improvements in the QOL questionnaires after VSB implantation. They stated that the better the speech understanding, the higher the QOL measure. Our results are consistent with this study. Considering the social and daily life limitations associated with hearing loss, the restoration of hearing capacity improves communication and prevents isolation.

Iwasaki et al. [25] reported on objective and subjective hearing abilities between patients with hearing aids and VSB round window vibroplasty. All patients were fitted with the same type of hearing aid before VSB surgery so they could compare audiological and QOL results with both devices. They obtained statistically significant results on audiological tests as well as the QOL questionnaires. Comparing satisfaction between conventional hearing aids and the VSB placed on the oval or round window, Atas et al. [40] observed better results with the VSB. They suggested that patient preference for the VSB could be attributed to the loss of ossicular chain sound transmission associated with previous middle ear surgery that worsens conventional hearing aid results when compared with the VSB.

High preoperative air conduction levels at 250 Hz in people over 60 years of age at implantation have been suggested as potentially negative factors in predicting subjective satisfaction with the VSB [41]. Our results are not exactly comparable to those of Han et al. [41] because in their study, all patients underwent incus vibroplasty and the QOL questionnaire was a numeric rating scale completed over the telephone. Most of our patients underwent round window vibroplasty, but we did not find significant QOL-related differences in our groups of <60 y and ≥60 y patients. All of them are daily implant users, some for more than ten years.

### 4.4. Limitations

A potential limitation of this study is the small number of participants, but both groups are homogenously distributed, and our results are similar to other published series.

### 4.5. Recommendations

A decline in long-term functional outcomes is possible in elderly populations due to age-related deterioration in bone conduction thresholds. Although we have not found audiological or QOL differences between our age groups, an earlier hearing decline in the ≥60 y group can be expected during follow-up even though age alone is not considered a limiting factor for VSB implantation.

Despite the knowledge that elderly people have worse bone conduction thresholds and that they will deteriorate over time, it is unpredictable when this will occur and how many years it will take to exceed the limits of VSB indication. VSB surgery has been demonstrated as a safe surgical procedure with no relevant complications. Successful outcomes are closely associated with stable preoperative bone conduction thresholds. To ensure the best possible results, it is critical to rule out any progressive sensorineural hearing loss prior to implantation [42].

In any case, VSB candidates with hearing thresholds close to the upper limit of the indication range, especially elderly candidates, should be informed of the higher risk for a potential lack of device performance later in the follow-up period. The hearing threshold deterioration in these cases may then put the patient in the cochlear implant indication range [42].

## 5. Conclusions

The VSB is an effective method of hearing restoration for conductive or mixed hearing loss. Age is not a limiting factor for VSB surgery. Unusual indications can also be considered in elderly patients.

## Figures and Tables

**Table 1:** Demographic and pre-implantation data.

n	<60 Years Old 11	≥60 Years Old 9	*p* Value
**Age** (years) (mean ± SD) (range)	45 ± 10 (24–56)	69 ± 7 (61–79)	<0.001
**Gender** (n) (%)			
Male	7 (64%)	5(56%)	
Female	4 (36%)	4(44%)	
**HL aetiology** (n) (%)			
Cholesteatoma	6 (55%)	2 (22%)	
Non-Cholesteatomatosus COM	3 (27%)	2 (22%)	
Previous surgery	2 (18%)	2 (22%)	
CSF leak	0	1 (11%)	
Paraganglioma	0	1 (11%)	
EAC stenosis	0	1 (11%)	
**PTA4_BC_** (dB)	28 ± 12	41 ± 11	0.018
**PTA4_AC_** (dB)	66 ± 15	73 ± 18	0.349
**ABG** (dB)	38 ± 10	32 ± 17	0.318
**SDS** (%)	19 ± 25	31 ± 43	0.297

HL hearing loss, SD standard deviation, COM chronic otitis media, CSF cerebrospinal fluid, EAC external auditory canal, PTA4_BC_ mean bone conduction threshold values at 500 Hz, 1000 Hz, 2000 Hz, and 4000 Hz, PTA4_AC_ mean pure-tone audiometry values at 500 Hz, 1000 Hz, 2000 Hz, and 4000 Hz, ABG air bone gap (PTA4_AC_ − PTA4_BC_), SDS speech discrimination score. PTA4_BC_, PTA4_SF_, ABG and SDS measured in the ear to be implanted.

**Table 2:** Post-implantation surgical data and audiometric outcomes.

	<60 Years Old	≥60 Years Old
Coupling option		
Round window	8 (73%)	7 (78%)
Incus short process	1 (9%)	1 (11%)
Stapes	2 (18%)	1 (11%)
mean ± SD		
PTA4_BC_ (dB)	33 ± 13	49 ± 11
PTA4_SF_ (dB)	34 ± 7	42 ± 9
pABG (dB)	1 ± 10	−6 ± 10
SDS (%)	95 ± 9	84 ± 14
Functional gain	32 ± 11	30 ± 15

SD standard deviation; PTA4_BC_ mean bone conduction threshold values at 500 Hz, 1000 Hz, 2000 Hz, and 4000 Hz; PTA4_SF_ mean sound field threshold values at 500 Hz, 1000 Hz, 2000 Hz, and 4000 Hz; pABG postoperative air bone gap (PTA4_SF_ − PTA4_BC_); SDS speech discrimination score; functional gain (preoperative PTA4_ac_ − PTA4_sf_). PTA4_BC_, PTA4_SF_, ABG, and SDS measured in the implanted ear.

**Table 3:** Mean Glasgow Benefit Inventory (GBI) score after implantation.

GBI Score	<60 Years Old Mean ± SD	≥60 Years Old Mean ± SD	*p* Value
Overall	61 ± 27	33 ± 24	0.05
General subscale	45 ± 10	23 ± 19	0.066
Social subscale	19 ± 41	14 ± 20	0.776
Physical health subscale	9 ± 35	−8 ± 14	0.328

## Data Availability

The data presented in this study are available on reasonable request from the corresponding author.
